# Therapeutic Role and Potential Mechanism of Resveratrol in Atherosclerosis: TLR4/NF-*κ*B/HIF-1*α*

**DOI:** 10.1155/2023/1097706

**Published:** 2023-05-31

**Authors:** Lin Guo, Xiaolu Zhang, Nuan Lv, Luming Wang, Jiali Gan, Xijuan Jiang, Yijing Wang

**Affiliations:** ^1^School of Integrative Medicine, Tianjin University of Traditional Chinese Medicine, 10 Poyanghu Road, West Area, Tuanbo New Town, Jinghai District, Tianjin 301617, China; ^2^School of Nursing, Tianjin University of Traditional Chinese Medicine, 10 Poyanghu Road, West Area, Tuanbo New Town, Jinghai District Tianjin 301617, China

## Abstract

Atherosclerosis, the main pathological basis of cardiovascular disease, is a chronic inflammatory disease that severely affects the quality of human life. Resveratrol (Res) is a natural polyphenol that is a major component of many herbs and foods. The present study analyzed resveratrol from the perspective of visualization and bibliometric analysis and found that resveratrol is closely related to the inflammatory response in cardiovascular diseases (associated with atherosclerosis). To explore the specific molecular mechanism of resveratrol, network pharmacology and Kyoto Encyclopedia of Genes and Genomes (KEGG) were used, in which HIF-1*α* signaling may be a key pathway in the treatment of AS. Furthermore, we induced the polarization of macrophage RAW264.7 to M1 type to generate inflammatory response by the combination of lipopolysaccharide (LPS) (200 ng/mL) + interferon-*γ* (IFN-*γ*) (2.5 ng/mL). LPS and IFN-*γ* increased the inflammatory factor levels of IL-1*β*, TNF-*α*, and IL-6 in RAW264.7, and the proportion of M1-type macrophages also increased, but the expression of inflammatory factors decreased after resveratrol administration, which confirmed the anti-inflammatory effect of resveratrol in AS. In addition, we found that resveratrol downregulated the protein expression of toll-like receptor 4 (TLR4)/NF-*κ*B/hypoxia inducible factor-1 alpha (HIF-1*α*). In conclusion, resveratrol has a significant anti-inflammatory effect, alleviates HIF-1*α*-mediated angiogenesis, and prevents the progression of AS through the TLR4/NF-*κ*B signaling pathway.

## 1. Introduction

**Figure 1:**
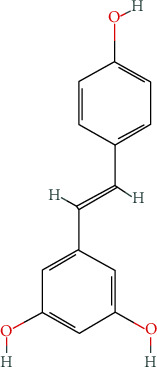
Chemical structure of resveratrol (PubChem CID: 445154).

Atherosclerosis is the primary pathological basis of cardiovascular disease (CVD) which is a key cause of mortality worldwide [1]. It is now well established that atherosclerosis is a chronic inflammatory disease with immune cells infiltrated [2, 3]. Furthermore, analyzing AS from a pathophysiological perspective, the progression of AS involves endothelial damage, abnormal lipid metabolism, and hemodynamic changes, and immunoreactive substances can be detected at each stage, demonstrating that AS is a chronic vascular inflammatory disease mediated by multiple risk factors [1, 4]. Macrophages are the sentinels of mammalian tissue homeostasis, intrinsic, and adaptive immunity [5, 6]. When endothelial cell (EC) is activated, a variety of inflammatory factors are expressed, such as interleukin (IL)-8, intercellular adhesion molecule-1 (ICAM-1), and vascular adhesion molecule-1 (VCAM-1), and, influenced by their microenvironment, monocyte precursors are generated. This is followed by differentiation into macrophages, in addition to accumulated lipoproteins or inflammatory factors. The initial motivation of macrophages may beneficial, but the conversion of phagocytosed oxidized low-density lipoprotein (ox-LDL) into foam cells eventually leads to a massive accumulation of foam cells, promoting plaque formation and exacerbating AS. Macrophages are highly plastic, and the combination of lipopolysaccharide (LPS), interferon-*γ* (IFN-*γ*), and stimulation with interleukin (IL)-4 is the most commonly used activation methods to induce macrophage polarization towards the M1 type [7]. Resveratrol is a natural polyphenol expressed within a wide range of plants and is found in 72 species of plants including mulberries and grapes [7, 8]. Resveratrol was first noticed in 1992 for the protective effects of red wine on the heart [9]. Since then, research on resveratrol has intensified, with numerous studies confirming the benefits of resveratrol on chronic diseases, including CVD, diabetes, Alzheimer's disease, and various cancers [10]. And resveratrol has diverse biological activities, including antioxidant, anti-inflammatory, antiproliferative, antiaging, and vascular modulating properties [11] (molecular formula of resveratrol Figure 1).

Bibliometrics is an effective subject evaluation method based on quantitative analysis and citation analysis of article topics, showing the overall development of a subject and the internal connections between related subjects [12]. Network pharmacology is a systematic and comprehensive study of the mechanisms of drugs which can assess pharmacological effects from biological aspect. Based on a network of drug-disease mechanism analysis, the relevant mechanisms for multitargeted drug treatment of diseases can be obtained [13].

**Figure 2:**
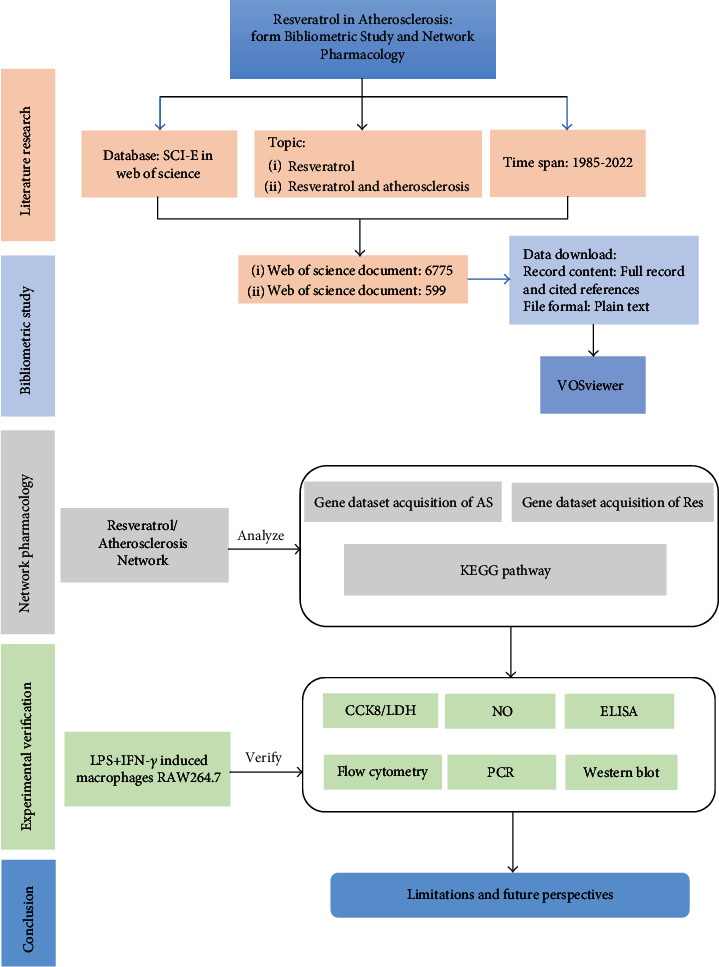
The flow chart of resveratrol in atherosclerosis.

In this study, the bibliometric method and network pharmacology were used to analyze the resveratrol, and the cluster and therapeutic target genes of resveratrol on AS were systematically analyzed. The results suggested that resveratrol exerted its anti-AS mechanism by inhibiting the inflammatory response. Furthermore, lipopolysaccharide- (LPS-) induced RAW264.7 model was constructed for validation. The specific process is shown in Figure 2.

## 2. Material and Methods

### 2.1. Material

RAW264.7 (ATCC number: TIB-71) were purchased from Hunan Fenghui Biotechnology Co., Ltd. Resveratrol (SR8070) and LPS (L8880) were purchased from Solarbio Technology Co., Ltd. (Beijing, China). CoCl_2_ was purchased from Sigma Company. IFN-*γ* (315-05) was purchased from PeproTech Company in the United States. ELISA kits of TNF-*α*, IL-1*β*, and IL-6 (SEA133Mu, SEA563Mu, and SEA079Mu) were purchased from Wuhan Cloud-Clone Company. Primer was purchased from Shengong Biotechnology Co., Ltd. (Shanghai, China). The LDH kit (A020-2-2) was purchased from Nanjing Jiancheng Biotechnology Co., Ltd. FITC-CD11c and PE-CD206 (11-0114-82, 12-2061-82) antibody were purchased from Thermo Fisher Scientific Co., Ltd. The antibody of TLR4, HIF-1*α*, and P-NF-*κ*B p65 (ab13556, ab239366, and ab32536) was purchased from Abcam in the UK.

### 2.2. Data and Methods for Bibliometrics

“Resveratrol AND Disease” and “Resveratrol AND Atherosclerosis”, all as the topic, were searched in the Web of Science core collection database until November 1, 2022, and the timespan was 1985-2022. The main data extracted included keywords, literature titles, and references. And data was analyzed using VOS viewer [14].

### 2.3. Gene Dataset Acquisition of Resveratrol and Atherosclerosis

Genes of resveratrol were gathered from the databases: Swiss Target Prediction Database (http://www.swisstargetprediction.ch) and the Similarity Ensemble Approach (SEA) Database (https://sea.bkslab.org/). With “Atherosclerosis” as the keyword, genes of atherosclerosis were gathered from the databases: the Online Mendelian Inheritance in Man (OMIM, http://www.omim.org/), GeneCards (https://www.genecards.org/), and DrugBank Database (https://go.drugbank.com), and just “Homo sapiens” proteins linked to atherosclerosis were selected.

### 2.4. KEGG Pathway Analysis

DAVID Bioinformatics Resources (https://david.ncifcrf.gov) is an integrated biological knowledge base containing large volumes of genes and proteins and extracting meaningful biological information that can be analyzed online, including Kyoto Encyclopedia of Genes and Genomes (KEGG) pathway enrichment analysis. The effective target proteins of resveratrol and AS were imported into the DAVID database for KEGG pathway enrichment analysis, and “Homo sapiens” was used as the selected target to obtain the effective functions and important pathways of resveratrol for the treatment of AS.

### 2.5. Cell Culture and Treatments

The RAW264.7 cell line (ATCC No. TIB-71) in this study was provided by Fenghui Biotechnology (Hunan Province, China). Resveratrol (Res) was purchased from Beijing Solarbio Technology (Lot SR8070). The cells were cultured in DMEM high sugar medium (Gibco, Lot C11995500BT) containing 10% fetal bovine serum (FBS, BI Biotechnology, Lot 04-001-1ACS) and 1% mixture of penicillin and streptomycin, placed in a cell culture incubator at 37°C with 5% CO_2_, and regularly changed and passaged, and logarithmic growth phase cells were taken for experiments. The control group, model group, Res high-dose group (10 *μ*mol/L), Res medium-dose group (5 *μ*mol/L), and Res low-dose group (1 *μ*mol/L) were set up (the dose concentrations were obtained by reference to the literature) [15]. The protective effect of resveratrol on the inflammatory response was investigated through the combination of LPS (200 ng/mL, China Solarbio Technology, Lot. L8880) + IFN-*γ* (2.5 ng/mL, PeproTech, USA, Lot. 315-05) to induce macrophages for 12 h to build the inflammation model and to verify whether resveratrol (10 *μ*mol/L) acts through HIF-1*α* to attenuate the inflammatory response using the HIF-1*α* agonist CoCl_2_ (100 *μ*mol/L, Sigma).

### 2.6. CCK-8 Assay for Macrophage Viability

RAW264.7 cells were inoculated in 96-well plates at a density of 1.5 × 10^5^ cells/mL in a well volume of 100 *μ*L. After 24 h incubation, the supernatant was discarded and 12 h synchronized. After 2 h of administration according to the above experimental groups, LPS + IFN-*γ* was added to induce inflammation. After further 12 h incubation, 10 *μ*L of CCK8 solution was added to each well, and the OD value at 450 nm was measured. The calculation formula is as follows: cell survival rate = [(As − Ab)/(Ac − Ab)] × 100% (As: the experimental group, Ac: control group, Ab: blank group).

### 2.7. LDH Efflux Assay to Detect Cellular Damage

The procedure was as described in Section 2.6. The LDH level in the cell supernatant was measured according to the instructions of the LDH Assay Kit (Nanjing Jiancheng Biotechnology, Lot: A020-2-2). The calculation formula is as follows: LDH content = (measured well OD − control well OD)/(average standard well OD − average blank well OD) × 200.

### 2.8. Flow Cytometry Detects M1 Polarization in Macrophages

The procedure was as described in Section 2.6. Digested cells were transferred to 1.5 mL centrifuge tubes, centrifuged and washed in PBS. Cells were washed three times in PBS and then labelled with FITC-CD11c marker, PE-CD206 (11-0114-82, 12-2061-82, Thermo Fisher Scientific Co., Ltd), at 4°C for 30 min. The labelled cells were analyzed using a flow cytometer.

### 2.9. ELISA Detects Inflammatory Factor Levels

The procedure was as described in Section 2.6. The supernatant was collected, and the levels of cellular inflammatory factors IL-1*β*, TNF-*α*, and IL-6 of each group were measured in ELISA kit (Wuhan Cloud-Clone Company, China).

### 2.10. Detection of NO Content

The procedure was as described in Section 2.6. The supernatant was collected, and the NO content in the supernatant was measured with the NO assay kit, and the OD value was detected at 540 nm.

### 2.11. Quantitative Real-Time PCR Analysis

RAW264.7 cells were inoculated in 12-well plates at a density of 1.5 × 10^5^ cells/mL in a well volume of 2000 *μ*L. The procedure was as described in Section 2.6. Trizol reagent was added to each well to extract total cellular RNA. cDNA was obtained by inversion and used as a template for real-time PCR analysis (RCP reaction system 20 *μ*L, reaction conditions: predenaturation at 95°C for 10 min, denaturation at 95°C for 15 s, annealing at 60°C for 1 min, 40 cycles). Statistical analysis of the data was performed using the 2^−ΔΔCT^ method, and the internal reference was GAPDH (the primer sequences are shown in Table 1).

### 2.12. Western Blot Analysis

Digest the cells, collect the cell suspension, and transfer to a 1.5 mL centrifuge tube, centrifuge and discard the supernatant. Proteins of cells were extracted using RIPA lysis buffer for 30 minutes. Equal amounts of protein lysates were transferred to PVDF membrane. After blocking with milk for 2 h, antibodies were added to identify protein level, at 4°C overnight. The secondary antibody incubation solution was incubated for 2 h at room temperature in a shaker, then washed 3 times. The PVDF membrane was placed in ECL developing solution.

### 2.13. Statistical Analysis

Using SPSS 22.0, the data was expressed as means ± SEM, and the differences between groups were compared using one-way ANOVA and graphing with GraphPad 8.0. *P* < 0.05 was a significant difference, and *P* < 0.01 is a very significant difference.

## 3. Results

### 3.1. Bibliometric Analysis of Resveratrol and Atherosclerosis

**Figure 3:**
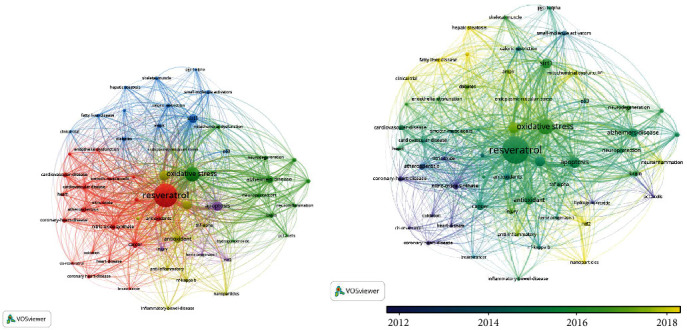
Bibliometric analysis of resveratrol and atherosclerosis: (a) bibliometric analysis of resveratrol and (b) bibliometric analysis of resveratrol and atherosclerosis.

“Resveratrol AND Disease” as the topic was for searched 6775 articles, with 50 times as inclusion criteria, and involved 246 topic terms (Figure 3(a)). “Resveratrol AND Atherosclerosis” as the topic was searched for 599 articles, with 20 times as inclusion criteria, and involved 50 topic terms (Figure 3(b)). As shown in Figure 3(a), the red cluster is the first dominant cluster (resveratrol, cardiovascular disease, atherosclerosis, coronary heart disease, heart, heart disease, etc.), highlighting the importance of resveratrol, which can be inferred to be closely related to cardiovascular disease and atherosclerosis. Atherosclerosis is an inflammatory condition in which endothelial injury predisposes to endothelial lipid accumulation and plaque formation [16]. Studies have shown that resveratrol regulated endothelial function in various ways, such as impaired vasorelaxation, leukocyte adhesion, aging, and mesenchymal transition [17]. Besides, resveratrol regulated the atherosclerotic lesion area by regulating blood lipids, including total cholesterol, triglyceride, and low-density lipoprotein [18–20]. Also, it inhibited the expression of matrix metalloproteinase-9, CD40 ligand (CD40L), and inflammatory factors [18]. Similarly, resveratrol regulated lipid metabolism and inhibited inflammation development through the TGF/ERK signaling pathway, playing a beneficial role in AS [21]. As shown in Figure 3(b), the red cluster was the first dominant cluster of resveratrol and atherosclerosis (resveratrol, cardiovascular disease, atherosclerosis, coronary heart disease, and antioxidant) that were associated with resveratrol's role as an antioxidant in AS. The second cluster (macrophage, inflammation, endothelial cells, smooth-muscle-cells, NF-kappa-b, etc.) found that resveratrol on AS was closely related to anti-inflammatory effects, and NF-kappa-b pathway played an important role.

### 3.2. Potential Target Genes and Network Analysis

**Figure 4:**
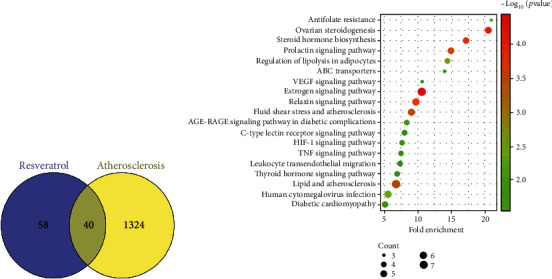
Network pharmacology of resveratrol and atherosclerosis: (a) overlapping genes of resveratrol and atherosclerosis and (b) KEGG pathway analysis on the 40 overlapping genes.

Swiss Target retrieved 69 candidate target genes, and SEA Target retrieved 78 candidate target genes, and a total of 98 resveratrol candidate target genes was identified (Supplementary Table 1: The 98 target genes of resveratrol). Based on the Disease Gene database, with 1276 in GeneCards, 100 is in DrugBank, and 215 is in OMIM. A total of 1364 genes associated with AS were identified after remove duplicates (Supplementary Table 2: The 1364 genes associated with AS). Forty genes were identified by matching potential targets of resveratrol and AS (Figure 4(a)). To further analyze the possible pharmacological mechanisms involved in AS and resveratrol, we performed KEGG pathway analysis on the 40 genes and retrieved 43 significant pathways (*P* < 0.05). Of these, 20 significant pathways were shown in Figure 4(b). The main mechanism of resveratrol in AS was closely related to the anti-inflammatory response, and the HIF-1 pathway was the most significant pathway.

### 3.3. Experimental Validation

#### 3.3.1. Resveratrol Inhibited the Inflammatory Response in RAW264.7 Cells

**Figure 5:**
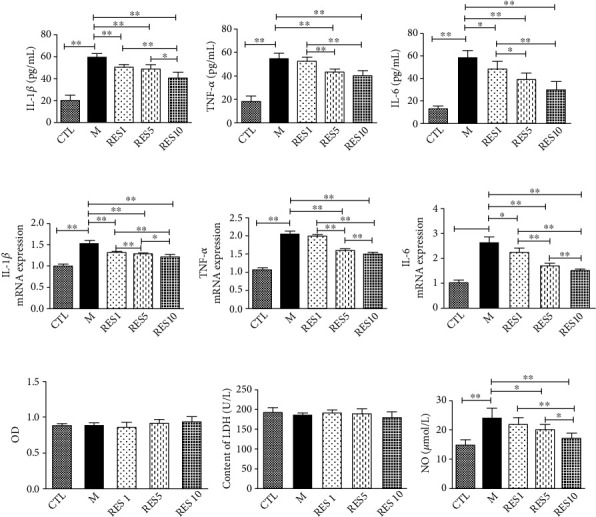
Resveratrol inhibited the inflammatory response in RAW264.7 cells. (a–c) The release of IL-1*β*, TNF-*α*, and IL-6 in cell supernatant was measured by ELISA kits. (d–f) The mRNA levels of IL-1*β*, TNF-*α*, and IL-6. (g) Cellular viability for RAW264.7 cells. (h) The release of LDH. (i) NO expression. Data were presented as mean ± SD. ^∗^*P* < 0.05; ^∗∗^*P* < 0.01.

Resveratrol can reduce the inflammatory response. When LPS + IFN-*γ* acted on macrophages, the cells were stimulated to develop an inflammatory response, and the levels of inflammatory factors IL-6, TNF-*α*, and IL-1*β* were significantly increased. However, this phenomenon was reversed by the resveratrol, and the best effect was seen in Res high-dose group (10 *μ*mol/L) [15]. The CCK8 and LDH results indicated the viability of the cells and the degree of damage, and the small differences between the components suggested less damage to the cells. LPS + IFN-*γ*-induced cells elevated NO levels and decreased by resveratrol intervention (Figure 5).

#### 3.3.2. Resveratrol Inhibited Macrophage Polarization towards M1 Type

**Figure 6:**
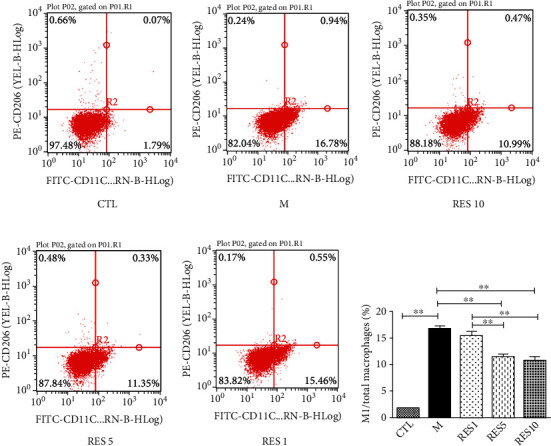
Effects of resveratrol on the M1 type macrophage. (a–f) Flow cytometric analysis of M1 macrophages. Data were presented as mean ± SD. ^∗^*P* < 0.05; ^∗∗^*P* < 0.01.

Resveratrol inhibited M1 polarization of macrophages and CD206 as a marker for the M1 type macrophages. Flow cytometry analysis showed a significantly higher proportion of M1 type macrophages with LPS + IFN-*γ*-induced and a reversal of the result after resveratrol intervention, in a drug concentration-dependent manner (Figure 6).

#### 3.3.3. Resveratrol Inhibited the Expression of TLR4, NF-*κ*B, and HIF-1*α*

**Figure 7:**
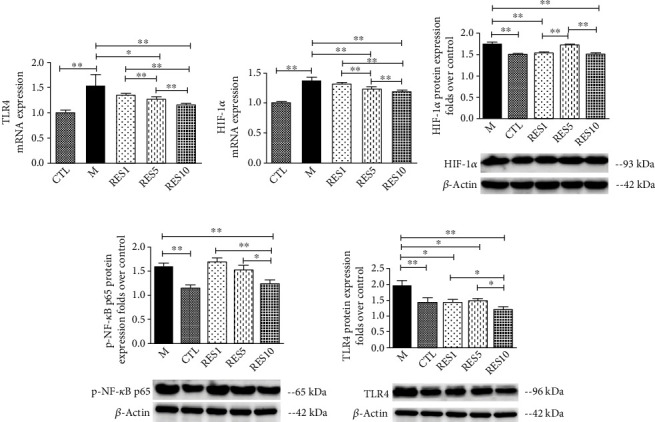
Resveratrol inhibited the expression of TLR4, NF-*κ*B, and HIF-1*α*. (a, b) The mRNA levels of TLR4 and HIF-1*α*. (c–e) The protein levels of TLR4, p-NF-*κ*B p65, and HIF-1*α*. Data were presented as mean ± SD. ^∗^*P* < 0.05; ^∗∗^*P* < 0.01.

Based on bibliometrics, network pharmacology, and experimental validation, resveratrol was found to reduce the inflammatory response. The mRNA expression of TLR4 and HIF-1*α* was significantly upregulated in macrophages after LPS + IFN-*γ* induced, and resveratrol reversed the phenomenon. And protein levels were verified to be consistent, with resveratrol reduced TLR4, p-NF-*κ*B p65, and HIF-1*α* protein expression (Figure 7).

#### 3.3.4. HIF-1*α* Is the Key for Resveratrol to Inhibit the M1 Type Macrophage

**Figure 8:**
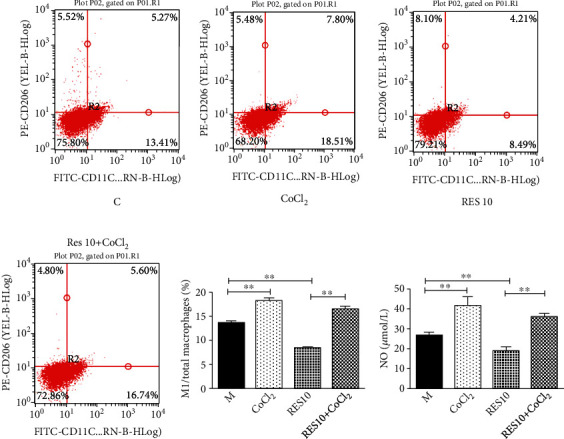
Effect of CoCl_2_ and resveratrol on M1 type macrophages. (a–e) Flow cytometric analysis of M1 macrophages. (f) NO expression. Data were presented as mean ± SD. ^∗^*P* < 0.05; ^∗∗^*P* < 0.01.

CoCl_2_, an agonist of HIF-1*α*, enhanced the expression of HIF-1*α* and reversed the effect of resveratrol on macrophages. Flow cytometry verified that CoCl_2_ induced a significant upregulation of the proportion of M1 macrophages. And resveratrol had a mitigating effect on M1 macrophages, which was led by CoCl_2_ induced. In addition, NO expression was followed by a similar phenomenon (Figure 8).

#### 3.3.5. Effect of CoCl_2_ and Resveratrol on Inflammatory Factors

**Figure 9:**
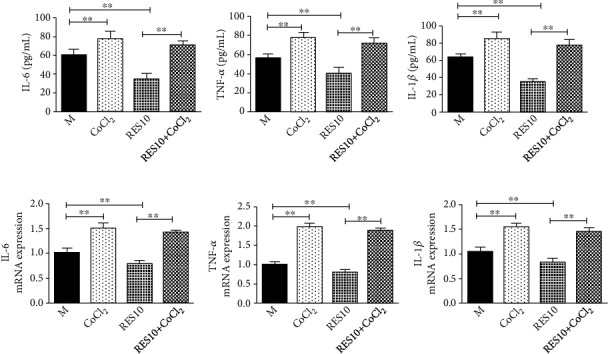
Effect of CoCl_2_ and resveratrol on inflammatory factors. (a–c) The release of IL-6, TNF-*α*, and IL-1*β* in cell supernatant was measured by ELISA kits. (d–f) The mRNA levels of IL-6, TNF-*α*, and IL-1*β*. Data were presented as mean ± SD. ^∗^*P* < 0.05, ^∗∗^*P* < 0.01.

The expression of HIF-1*α* led to an increase of inflammatory factors as well. And CoCl_2_ counteracted the anti-inflammatory effect of resveratrol and suggested that HIF-1*α* was the key target of resveratrol, which reduced the inflammatory response (Figure 9).

## 4. Discussion and Conclusion

AS is a serious health problem with high morbidity and high mortality worldwide, and its incidence increases with age [22]. In terms of physiopathology, the pathogenic mechanisms of AS are complex. AS is a lipid-driven arterial disease, but it is always accompanied by chronic nonresolving inflammation [23]. Macrophages play an important role in the inflammatory response and are the key immune cells closely involved in the pathogenesis of AS and are important targets for AS diagnosis and new therapies [24]. Various triggers of AS promote the accumulation of M1 type macrophages in the coronary arteries, which cannot be digested to form foam cells, leading to plaque formation [25, 26]. Therefore, there is an urgent need to find more effective therapeutic strategies to suppress the inflammatory response, gain insight into the pathogenic mechanisms, and curb the progression of AS. Bibliometrics is the analysis of the literature and its measurement characteristics to predict the current trends of a field [27]. In this study, a literature search and analysis were conducted using resveratrol and AS as the subject term and found that resveratrol played an important anti-inflammatory role in AS. Network pharmacology is a new approach to elucidate the mechanisms of drugs in disease [13]. In this study, 98 resveratrol-associated genes and 1364 AS-associated genes and 40 overlapping genes were identified by network pharmacology [13]. To gain a deeper effects of resveratrol on AS, we performed KEGG pathway enrichment and obtained 43 pathways, of which the top 20 pathways were mainly associated with inflammatory responses, and the important one involved was HIF-1 pathway. In addition, our subsequent experiments showed that resveratrol reduced the inflammation in AS by inhibiting HIF-1*α*, an important target in the HIF-1 pathway, NF-*κ*B, and TLR4.

A growing body of research suggests that resveratrol plays an important role in the treatment of the inflammatory response to AS [20, 28]. In this study, we found that resveratrol and AS targets were closely associated with inflammation and that the HIF-1 pathway played an important role. Resveratrol was showed to exert anti-inflammatory effects in AS by inhibiting bisphenol A (BPA), elevated sirtuin 1, and Nijmegen breakage syndrome 1 (NBS1) expression [29]. And sirtuin 1 inhibited nuclear translocation of NF-*κ*B and reduced hyaluronate synthase 2 expression [30]. Resveratrol can also inhibit the inflammatory response to AS by directly targeting IL-1*β* [31]. TLR4 is a typical pattern recognition receptor in the intrinsic immune response, which induces the recruitment of leukocytes to smooth muscle cells and the release of inflammatory factors such as IL-6 [32, 33]. However, resveratrol can inhibit the TLR4 pathway. NF-*κ*B is an important nuclear transcription factor in the inflammatory response, mediated the transcription of inflammatory factors, and released large amounts of inflammatory factors, and NF-*κ*B is also influenced by TLR4 [34, 35]. In addition to the primary role of macrophages in the AS inflammatory response, adhesion of monocytes to ECs also produced inflammatory factors [2, 36]. Studies have demonstrated that resveratrol attenuates the TNF-*α*-induced degradation of I*κ*B-*α* and nuclear translocation of NF-*κ*B p65 by inflammatory factors and reduces macrophage expression in the aorta [37–40]. Thus, resveratrol's inhibition of TLR4 also affects NF-*κ*B, thereby inhibiting the inflammatory response [41, 42].

HIF-1 was found to be an important pathway by KEGG analysis. During the development of AS, extracellular matrix, lipids, and macrophages generate plaques, indirectly appear hypoxic areas, and activate the HIF-1 pathway and its associated factors. HIF-1 consists of HIF-1*β* and the oxygen-sensitive HIF-1*α*, which enter the nucleus to initiate a series of translational expressions in hypoxic plaque tissue and plaques [43]. Although HIF-1*α* also has some dominant functions, within macrophages, it is mainly involved in inflammatory responses, angiogenesis, and metabolic reprogramming [44]. The expression of HIF-1*α* also inhibits the expression of the transporter protein ABCA1 and ApoA1, preventing macrophages from removing excess lipids and exacerbating hypoxia and inflammatory responses [45–47].

The two most important factors in AS are dyslipidemia and inflammatory response [48, 49]. Most of the top 20 pathways obtained from the KEGG analysis were closely linked to the inflammatory response, excluding most hormones with inflammatory responses, such as prolactin, which inhibited TLR-4/NF-*κ*B pathway and reduced the expression of inflammatory cytokines [50, 51]. Relaxin downregulated TLR4 expression and increased M2 type macrophages, exerting a protective effect [52]. It is also enriched in lipid and AS-related pathways, and resveratrol has been shown to have pharmacological effects in preventing LDL oxidation in AS [53]. Resveratrol was showed to improve low shear stress and oxidative stress to treat AS [54]. Letter pathways such as lipolysis [55], human cytomegalovirus infection [56], diabetic complication-related pathways [57, 58], and C-type lectin receptor signaling pathway are all closely related to the inflammatory response [59]. In response to the aforementioned pathways, resveratrol inhibited adipogenesis in mice on a high-fat diet, promoting lipolysis in adipose tissue [60, 61], and inhibited viral induced activation of epidermal growth factor receptor (EGFR), significantly reducing human cytomegalovirus DNA replication [62, 63]. Additionally, a large number of studies confirmed that resveratrol can reduce insulin resistance and played a beneficial role in chronic inflammation in diabetic patients [64, 65]. In THP-1 cells and RAW264.7 cells, resveratrol inhibited bacterial phagocytosis by macrophages by downregulating the expression of C-type lectin receptor [66]. The HIF-1 pathway was screened as a major pathway, and although, it was well studied in terms of inflammatory responses [67, 68]. It has proved that resveratrol and omega-3 can significantly improve the histopathological damage of atherosclerosis in vivo [69], but studies of resveratrol's anti-inflammatory effects via the HIF-1 pathway are extremely rare and even less in AS. Therefore, our results showed that resveratrol inhibited the inflammatory response by modulating the TLR4/NF-*κ*B/HIF-1*α* pathway, suggesting that resveratrol exerted a protective effect against AS through its anti-inflammatory effects.

In summary, resveratrol is a drug with multitarget and multipathway and especially plays an important anti-inflammatory role in the inflammatory response, and it is expected to be a therapeutic or preventive drug for AS. Bibliometric study and network pharmacology of resveratrol revealed that the key pathway through anti-inflammatory was closely related to the TLR4/NF-*κ*B/HIF-1*α* pathway. Further validation of other pathways is an important way to explore the mechanisms associated with resveratrol.

## Figures and Tables

**Table 1:** Primer sequences for PCR.

Name	Sequence (5′ to 3′)		Length/bp
IL-1*β*	F	ACCCTCACACTCACAAACCA	246
	R	GGCAGAGAGGAGGTTGACTT	
IL-6	F	AGACTTCCATCCAGTTGCCT	113
	R	CAGGTCTGTTGGGAGTGGTA	
TNF-*α*	F	CCACCACGCTCTTCTGTCTA	118
	R	TGGTTTGTGAGTGTGAGGGT	
TLR4	F	AGGCAGCAGGTGGAATTGTA	174
	R	GGTCCAAGTTGCCGTTTCTT	
HIF-1*α*	F	AGGTGGAGGAGCTGTTGTAC	184
	R	CTCTGCTCATCATCCGACCT	

## Data Availability

All data generated or analyzed during this study are included in this published article.

## Supplementary Materials

Supplementary Materials The supplementary material for this article can be found. Supplementary Table 1: the identified 98 potential targets of resveratrol. Supplementary Table 2: the identified 1364 genes relevant to atherosclerosis.
