# Supplementary material for: Therapeutic Role and Potential Mechanism of Resveratrol in Atherosclerosis: TLR4/NF-κB/HIF-1α
Source: Mediators Inflamm. 2023 May 31;2023:1097706. doi: 10.1155/2023/1097706 (PMC10247328; doi:10.1155/2023/1097706)
Supplement: Supplementary Materials — The supplementary material for this article can be found. Supplementary Table 1: the identified 98 potential targets of resveratrol. Supplementary Table 2: the identified 1364 genes relevant to atherosclerosis. [file 1097706.f1.zip › Table_1(Drug target).docx]

| NO. | Gene names |
| --- | --- |
| 1 | AHR |
| 2 | NFE2L2 |
| 3 | APP |
| 4 | TRPA1 |
| 5 | TTR |
| 6 | CA3 |
| 7 | NQO2 |
| 8 | CA5B |
| 9 | CA6 |
| 10 | CA5A |
| 11 | CYP1B1 |
| 12 | PTGS1 |
| 13 | CA14 |
| 14 | CA7 |
| 15 | ESR1 |
| 16 | CA15 |
| 17 | CA13 |
| 18 | CA4 |
| 19 | PTGS2 |
| 20 | MAOA |
| 21 | CA12 |
| 22 | CA9 |
| 23 | CA1 |
| 24 | CA2 |
| 25 | GFER |
| 26 | CYP1A2 |
| 27 | CYP3A4 |
| 28 | PIK3CB |
| 29 | CYP2C9 |
| 30 | SLC6A2 |
| 31 | DPP4 |
| 32 | CYP2C19 |
| 33 | PIK3CA |
| 34 | LSDA |
| 35 | TUBB1 |
| 36 | ALDH5A1 |
| 37 | SSSIM |
| 38 | CA |
| 39 | ABAT |
| 40 | SCP-2 |
| 41 | RELA |
| 42 | AKR1B1 |
| 43 | PTBB |
| 44 | AKR1B10 |
| 45 | IMA1 |
| 46 | MIF |
| 47 | HSD17B3 |
| 48 | HSD11B1 |
| 49 | CISD1 |
| 50 | KCNK2 |
| 51 | PIGA |
| 52 | ALOX5 |
| 53 | ODC1 |
| 54 | GLO1 |
| 55 | ALOX15B |
| 56 | PPO2 |
| 57 | MAPT |
| 58 | POL |
| 59 | ESR2 |
| 60 | G6PC |
| 61 | CXCL12 |
| 62 | ABCG2 |
| 63 | BACE1 |
| 64 | CYP1A1 |
| 65 | F3 |
| 66 | MMP9 |
| 67 | LCK |
| 68 | SYK |
| 69 | CYP19A1 |
| 70 | EGFR |
| 71 | TUBB3 |
| 72 | ABCB1 |
| 73 | MMP2 |
| 74 | CLK1 |
| 75 | PTK2B |
| 76 | ESRRA |
| 77 | ESRRB |
| 78 | IGF1R |
| 79 | ALOX15 |
| 80 | HSD17B2 |
| 81 | HSD17B1 |
| 82 | INSR |
| 83 | DYRK1A |
| 84 | DYRK1B |
| 85 | KIT |
| 86 | SRC |
| 87 | LTB4R |
| 88 | AR |
| 89 | ERN1 |
| 90 | HSD17B14 |
| 91 | HDAC2 |
| 92 | ABCC1 |
| 93 | HDAC8 |
| 94 | SHBG |
| 95 | CBR1 |
| 96 | CYP11B1 |
| 97 | CYP11B2 |
| 98 | CYP17A1 |
