# Supplementary material for: Therapeutic Role and Potential Mechanism of Resveratrol in Atherosclerosis: TLR4/NF-κB/HIF-1α
Source: Mediators Inflamm. 2023 May 31;2023:1097706. doi: 10.1155/2023/1097706 (PMC10247328; doi:10.1155/2023/1097706)
Supplement: Supplementary Materials — The supplementary material for this article can be found. Supplementary Table 1: the identified 98 potential targets of resveratrol. Supplementary Table 2: the identified 1364 genes relevant to atherosclerosis. [file 1097706.f1.zip › Table_2(Disease target).docx]

| NO. | Gene names |
| --- | --- |
| 1 | AHR |
| 2 | NFE2L2 |
| 3 | APP |
| 4 | TTR |
| 5 | PTGS1 |
| 6 | ESR1 |
| 7 | PTGS2 |
| 8 | MAOA |
| 9 | CYP3A4 |
| 10 | PIK3CB |
| 11 | CYP2C9 |
| 12 | DPP4 |
| 13 | CYP2C19 |
| 14 | RELA |
| 15 | AKR1B1 |
| 16 | MIF |
| 17 | HSD11B1 |
| 18 | KCNK2 |
| 19 | ALOX5 |
| 20 | ALOX15B |
| 21 | MAPT |
| 22 | ESR2 |
| 23 | CXCL12 |
| 24 | ABCG2 |
| 25 | CYP1A1 |
| 26 | F3 |
| 27 | MMP9 |
| 28 | CYP19A1 |
| 29 | EGFR |
| 30 | ABCB1 |
| 31 | MMP2 |
| 32 | ALOX15 |
| 33 | INSR |
| 34 | SRC |
| 35 | LTB4R |
| 36 | AR |
| 37 | HDAC2 |
| 38 | ABCC1 |
| 39 | SHBG |
| 40 | CYP11B2 |
| 41 | APOE |
| 42 | ATHS |
| 43 | APOB |
| 44 | APOA1 |
| 45 | ABCA1 |
| 46 | LDLR |
| 47 | ABCG5 |
| 48 | PCSK9 |
| 49 | LMNA |
| 50 | LIPC |
| 51 | ABCG8 |
| 52 | LPL |
| 53 | CRP |
| 54 | PPARG |
| 55 | LPA |
| 56 | CCL2 |
| 57 | CETP |
| 58 | LCAT |
| 59 | APOA2 |
| 60 | IL6 |
| 61 | OLR1 |
| 62 | PON1 |
| 63 | CYP27A1 |
| 64 | ADIPOQ |
| 65 | SELE |
| 66 | ACE |
| 67 | INS |
| 68 | TLR4 |
| 69 | TNF |
| 70 | MTHFR |
| 71 | ELN |
| 72 | NOS3 |
| 73 | LDLRAP1 |
| 74 | SELP |
| 75 | ICAM1 |
| 76 | IL10 |
| 77 | APOC3 |
| 78 | ALB |
| 79 | VCAM1 |
| 80 | SERPINE1 |
| 81 | LIPA |
| 82 | HMGCR |
| 83 | SCARB1 |
| 84 | PLA2G7 |
| 85 | PPARA |
| 86 | CYP7A1 |
| 87 | WRN |
| 88 | IL1B |
| 89 | AGER |
| 90 | EDN1 |
| 91 | SPP1 |
| 92 | VWF |
| 93 | AGTR1 |
| 94 | DYNC2LI1 |
| 95 | CX3CR1 |
| 96 | APOA1-AS |
| 97 | ABCG1 |
| 98 | LEP |
| 99 | MPO |
| 100 | CD36 |
| 101 | APOA5 |
| 102 | LIPG |
| 103 | PLTP |
| 104 | VEGFA |
| 105 | THBD |
| 106 | APOC2 |
| 107 | ACTA2 |
| 108 | CELA2A |
| 109 | MYH11 |
| 110 | IL18 |
| 111 | AGT |
| 112 | CDKN2B-AS1 |
| 113 | EPHX2 |
| 114 | RETN |
| 115 | SMAD3 |
| 116 | MSR1 |
| 117 | LOX |
| 118 | TGFB2 |
| 119 | NAMPT |
| 120 | PLAT |
| 121 | PON2 |
| 122 | HSPD1 |
| 123 | FBN1 |
| 124 | CXCL8 |
| 125 | PON3 |
| 126 | SMARCA4 |
| 127 | MIR6886 |
| 128 | ENG |
| 129 | MMP3 |
| 130 | HMOX1 |
| 131 | GHR |
| 132 | SOAT1 |
| 133 | CD40 |
| 134 | CYBA |
| 135 | TGFBR2 |
| 136 | COL3A1 |
| 137 | SMAD2 |
| 138 | FABP4 |
| 139 | TGFB3 |
| 140 | TGFBR1 |
| 141 | MIR126 |
| 142 | TGFB1 |
| 143 | BGN |
| 144 | MGP |
| 145 | NIPSNAP3B |
| 146 | LDLR-AS1 |
| 147 | ABCC6 |
| 148 | ERCC6 |
| 149 | LOC106560211 |
| 150 | ZMPSTE24 |
| 151 | KNG1 |
| 152 | NOS2 |
| 153 | PNPLA2 |
| 154 | MYLK |
| 155 | PPP1R17 |
| 156 | REN |
| 157 | SMPD1 |
| 158 | SMAD4 |
| 159 | TLR2 |
| 160 | PRKG1 |
| 161 | MIR145 |
| 162 | PECAM1 |
| 163 | APOH |
| 164 | ENPP1 |
| 165 | DOCK6 |
| 166 | LTA |
| 167 | CAT |
| 168 | PSEN1 |
| 169 | IRAK1 |
| 170 | GPIHBP1 |
| 171 | XYLT1 |
| 172 | MIR155 |
| 173 | ITGB3 |
| 174 | CST3 |
| 175 | ACTA2-AS1 |
| 176 | XYLT2 |
| 177 | MIR21 |
| 178 | TNNT2 |
| 179 | TP53 |
| 180 | MAT2A |
| 181 | MFAP5 |
| 182 | HEY2 |
| 183 | FOXE3 |
| 184 | CD68 |
| 185 | SLC2A10 |
| 186 | HLA-DRB1 |
| 187 | TP53COR1 |
| 188 | TNFRSF11B |
| 189 | CD14 |
| 190 | F5 |
| 191 | TUG1 |
| 192 | F2 |
| 193 | SMARCAL1 |
| 194 | TNFSF4 |
| 195 | MIR146A |
| 196 | JAK2 |
| 197 | H19 |
| 198 | KCNJ5 |
| 199 | ERCC1 |
| 200 | NDE1 |
| 201 | SRFBP1 |
| 202 | LOC113939944 |
| 203 | F7 |
| 204 | NPC1 |
| 205 | SAA1 |
| 206 | F13A1 |
| 207 | APOA4 |
| 208 | EDNRA |
| 209 | NLRP3 |
| 210 | CD40LG |
| 211 | C4A |
| 212 | CX3CL1 |
| 213 | COG2 |
| 214 | SIRT1 |
| 215 | RNF213 |
| 216 | AKT1 |
| 217 | TNXB |
| 218 | MMP1 |
| 219 | NR1H4 |
| 220 | PTPN22 |
| 221 | ANGPTL3 |
| 222 | SOAT2 |
| 223 | FCGR2A |
| 224 | LRP1 |
| 225 | ERCC8 |
| 226 | IGF1 |
| 227 | CBS |
| 228 | SERPINC1 |
| 229 | IL1RN |
| 230 | GJA4 |
| 231 | SOD2 |
| 232 | IL17A |
| 233 | MIR17 |
| 234 | PIK3CG |
| 235 | SREBF1 |
| 236 | ITGAM |
| 237 | ALOX5AP |
| 238 | STAT3 |
| 239 | NPPB |
| 240 | BANF1 |
| 241 | MIR140 |
| 242 | TIMP1 |
| 243 | AAT1 |
| 244 | AAT2 |
| 245 | HP |
| 246 | MIR499A |
| 247 | HOXC-AS1 |
| 248 | NFIA-AS1 |
| 249 | IL4 |
| 250 | SERPINA3 |
| 251 | CAV1 |
| 252 | IFNG |
| 253 | NR1H2 |
| 254 | PPARD |
| 255 | HULC |
| 256 | TNNI3 |
| 257 | SORL1 |
| 258 | CLU |
| 259 | FGB |
| 260 | GGCX |
| 261 | CFH |
| 262 | GCLM |
| 263 | ALMS1 |
| 264 | HDAC9 |
| 265 | MIAT |
| 266 | SOD1 |
| 267 | GHRL |
| 268 | ACTC1 |
| 269 | MIR210 |
| 270 | PTX3 |
| 271 | SREBF2 |
| 272 | NBN |
| 273 | TNFAIP3 |
| 274 | JUN |
| 275 | ANGPTL4 |
| 276 | FGA |
| 277 | APOC1 |
| 278 | MYH7 |
| 279 | AHSG |
| 280 | CEL |
| 281 | CDKN2A |
| 282 | HMGB1 |
| 283 | LRP2BP-AS1 |
| 284 | TLR7 |
| 285 | CD5L |
| 286 | SCN5A |
| 287 | MTTP |
| 288 | CEP19 |
| 289 | DNASE1 |
| 290 | TRAF6 |
| 291 | PDE4D |
| 292 | STAT4 |
| 293 | PF4 |
| 294 | MIR195 |
| 295 | CSF1 |
| 296 | SOD3 |
| 297 | GP6 |
| 298 | AGTR2 |
| 299 | DNMT1 |
| 300 | GGT1 |
| 301 | CHIT1 |
| 302 | MMP8 |
| 303 | IL1A |
| 304 | CD163 |
| 305 | KLF4 |
| 306 | NPPA |
| 307 | MAPK14 |
| 308 | MIR208A |
| 309 | IGFBP3 |
| 310 | PSEN2 |
| 311 | GNB3 |
| 312 | SCARB2 |
| 313 | GPT |
| 314 | BRCC3 |
| 315 | ZNF687 |
| 316 | TGFBR3 |
| 317 | CCR5 |
| 318 | CCR2 |
| 319 | TNFRSF1A |
| 320 | FGF23 |
| 321 | MMP14 |
| 322 | NR1I2 |
| 323 | IL1R1 |
| 324 | TXN |
| 325 | TIMP2 |
| 326 | PLA2G2A |
| 327 | HIF1A |
| 328 | CDKN2B |
| 329 | LGALS2 |
| 330 | DCN |
| 331 | CCL5 |
| 332 | MMP12 |
| 333 | FOXP3 |
| 334 | TNFSF11 |
| 335 | TERT |
| 336 | CDH5 |
| 337 | GSTT1 |
| 338 | PAPPA |
| 339 | PDCD1 |
| 340 | ACE2 |
| 341 | SLC17A5 |
| 342 | PPBP |
| 343 | CXCR4 |
| 344 | PPARGC1A |
| 345 | BLK |
| 346 | HFE |
| 347 | GSTM1 |
| 348 | GCLC |
| 349 | NOX4 |
| 350 | THBS1 |
| 351 | SERPIND1 |
| 352 | ETS1 |
| 353 | ADRB2 |
| 354 | TOR2A |
| 355 | NFKB1 |
| 356 | MIR33A |
| 357 | SELPLG |
| 358 | MYH6 |
| 359 | NKX2-5 |
| 360 | GDF15 |
| 361 | MTRR |
| 362 | TCF7L2 |
| 363 | FCGR3B |
| 364 | UTS2 |
| 365 | CHEK2 |
| 366 | PGF |
| 367 | RAF1 |
| 368 | NOTCH1 |
| 369 | LCN2 |
| 370 | HGF |
| 371 | SGCB |
| 372 | SELL |
| 373 | SCAP |
| 374 | PTGIS |
| 375 | ECE1 |
| 376 | RHOA |
| 377 | MIR1306 |
| 378 | CCL13 |
| 379 | UCP2 |
| 380 | PSMA6 |
| 381 | KL |
| 382 | MAPK1 |
| 383 | CTLA4 |
| 384 | CDH13 |
| 385 | CASP3 |
| 386 | NPY |
| 387 | BGLAP |
| 388 | NFKB2 |
| 389 | MTOR |
| 390 | TFPI |
| 391 | NEXN |
| 392 | CHI3L1 |
| 393 | APLN |
| 394 | HAND2-AS1 |
| 395 | RSAD2 |
| 396 | CCL11 |
| 397 | LOC114803475 |
| 398 | IL2 |
| 399 | THSD1 |
| 400 | LIPE |
| 401 | ERCC4 |
| 402 | GPX1 |
| 403 | LGALS3 |
| 404 | COMT |
| 405 | FGG |
| 406 | CREB3L3 |
| 407 | TREM1 |
| 408 | S100A12 |
| 409 | TIMP3 |
| 410 | NR1H3 |
| 411 | KDR |
| 412 | C1QA |
| 413 | PLN |
| 414 | GUCY1A1 |
| 415 | RHOD |
| 416 | MTR |
| 417 | CYBB |
| 418 | NPC2 |
| 419 | ELANE |
| 420 | MIR92B |
| 421 | CES1 |
| 422 | TTN |
| 423 | VKORC1 |
| 424 | LRP8 |
| 425 | C3 |
| 426 | PRKCD |
| 427 | IGFBP1 |
| 428 | HSPA4 |
| 429 | SP1 |
| 430 | ADAMTS7 |
| 431 | FLT1 |
| 432 | SERPINA1 |
| 433 | ABCC9 |
| 434 | PLG |
| 435 | ANGPT2 |
| 436 | GSTA4 |
| 437 | MIR34A |
| 438 | PARP1 |
| 439 | VLDLR |
| 440 | ACVRL1 |
| 441 | F2R |
| 442 | MAPK3 |
| 443 | SLC10A2 |
| 444 | CAPN10 |
| 445 | BSG |
| 446 | GP1BA |
| 447 | ABCG4 |
| 448 | FGF21 |
| 449 | PDGFB |
| 450 | ITGB2 |
| 451 | CLEC4A |
| 452 | APBB1 |
| 453 | ANXA5 |
| 454 | AGXT |
| 455 | IRF5 |
| 456 | F8 |
| 457 | FN1 |
| 458 | XDH |
| 459 | ABCA4 |
| 460 | PHACTR1 |
| 461 | ALDH2 |
| 462 | BRCA1 |
| 463 | LTA4H |
| 464 | ITGA2 |
| 465 | EGR1 |
| 466 | CCN3 |
| 467 | FLNC |
| 468 | CTNNB1 |
| 469 | ADAMTS1 |
| 470 | DHCR7 |
| 471 | MFGE8 |
| 472 | ANTXR1 |
| 473 | HGD |
| 474 | ANGPTL6 |
| 475 | PTOV1 |
| 476 | STAP1 |
| 477 | ADAMTS4 |
| 478 | MIR29A |
| 479 | ADAM17 |
| 480 | NINJ1 |
| 481 | CXCL10 |
| 482 | MBL2 |
| 483 | IL33 |
| 484 | CSRP3 |
| 485 | FABP2 |
| 486 | RBP4 |
| 487 | KLF2 |
| 488 | VCL |
| 489 | MYOCD |
| 490 | XBP1 |
| 491 | PROC |
| 492 | NR3C1 |
| 493 | MIR182 |
| 494 | P2RY12 |
| 495 | SLC2A9 |
| 496 | USF1 |
| 497 | APOL1 |
| 498 | TAAR8 |
| 499 | FAM186A |
| 500 | LINC01362 |
| 501 | LINC00400 |
| 502 | LINC01361 |
| 503 | PMM2 |
| 504 | ALOX12 |
| 505 | NR3C2 |
| 506 | APOM |
| 507 | C4B |
| 508 | CYP7B1 |
| 509 | FOXO3 |
| 510 | MYBPC3 |
| 511 | HAND2 |
| 512 | NOS1 |
| 513 | STAT1 |
| 514 | MECP2 |
| 515 | BCL2 |
| 516 | NOS1AP |
| 517 | SOCS3 |
| 518 | CDKN1A |
| 519 | PTEN |
| 520 | CCN2 |
| 521 | PTH |
| 522 | TLR9 |
| 523 | C1R |
| 524 | CLCN1 |
| 525 | DNASE1L3 |
| 526 | GAS6 |
| 527 | NRG1 |
| 528 | LMF1 |
| 529 | TFEB |
| 530 | ANKRD1 |
| 531 | CASR |
| 532 | IL20 |
| 533 | CACNA1C |
| 534 | CP |
| 535 | AGXT2 |
| 536 | CXCL16 |
| 537 | MIR214 |
| 538 | AHSP |
| 539 | EDNRB |
| 540 | VDR |
| 541 | CR2 |
| 542 | PLA2G10 |
| 543 | FABP3 |
| 544 | PTPN11 |
| 545 | CTSL |
| 546 | CHST1 |
| 547 | MMP13 |
| 548 | IL2RA |
| 549 | BDNF |
| 550 | MIR30A |
| 551 | EGF |
| 552 | MIR142 |
| 553 | LPAL2 |
| 554 | MIR221 |
| 555 | CASP1 |
| 556 | PRL |
| 557 | MIR199A1 |
| 558 | SRF |
| 559 | MME |
| 560 | APCS |
| 561 | NOX1 |
| 562 | ITGB1 |
| 563 | SQSTM1 |
| 564 | MALAT1 |
| 565 | NFKBIA |
| 566 | ANGPT1 |
| 567 | DMD |
| 568 | DES |
| 569 | ARID5B |
| 570 | CXCL1 |
| 571 | SOCS1 |
| 572 | CCL3 |
| 573 | GCKR |
| 574 | CPE |
| 575 | LIPI |
| 576 | SPEG |
| 577 | CXCR2 |
| 578 | CD59 |
| 579 | SULT1A3 |
| 580 | FAS |
| 581 | SYNGR3 |
| 582 | GRAMD1C |
| 583 | FAM95C |
| 584 | KIAA0319L |
| 585 | LAMP2 |
| 586 | MIR196A2 |
| 587 | MIR143 |
| 588 | ITLN1 |
| 589 | ADD1 |
| 590 | CSF2 |
| 591 | COX5A |
| 592 | RAC1 |
| 593 | ADAMTSL1 |
| 594 | TNFRSF1B |
| 595 | VCAN |
| 596 | ATM |
| 597 | AKT2 |
| 598 | ACTA1 |
| 599 | ACTN2 |
| 600 | SIRT6 |
| 601 | IL12A |
| 602 | GSR |
| 603 | COL4A1 |
| 604 | PRTN3 |
| 605 | RYR3 |
| 606 | C5 |
| 607 | TAFAZZIN |
| 608 | PDGFRA |
| 609 | NR4A3 |
| 610 | MIR33B |
| 611 | SERPINA12 |
| 612 | RARRES2 |
| 613 | MAPK8 |
| 614 | RHBDF2 |
| 615 | HABP2 |
| 616 | TNFSF12 |
| 617 | PLAUR |
| 618 | CD4 |
| 619 | CTSA |
| 620 | PAOD1 |
| 621 | IL15 |
| 622 | GAS5 |
| 623 | SORT1 |
| 624 | CCDC71L |
| 625 | MIR19B1 |
| 626 | IRS2 |
| 627 | AOC3 |
| 628 | FBLN5 |
| 629 | GSTP1 |
| 630 | MIR144 |
| 631 | MIR223 |
| 632 | FOXO1 |
| 633 | IFNGR1 |
| 634 | CTH |
| 635 | TNFSF10 |
| 636 | MIR92A1 |
| 637 | IL6ST |
| 638 | DSP |
| 639 | TPM1 |
| 640 | DSG2 |
| 641 | SLC12A4 |
| 642 | TMPO |
| 643 | SGCD |
| 644 | EYA4 |
| 645 | BAG3 |
| 646 | TCAP |
| 647 | TMEM43 |
| 648 | TSFM |
| 649 | LDB3 |
| 650 | NDUFAF2 |
| 651 | FKTN |
| 652 | PPP1R13L |
| 653 | CASZ1 |
| 654 | CEP85L |
| 655 | RBM20 |
| 656 | TTN-AS1 |
| 657 | DSG2-AS1 |
| 658 | MHRT |
| 659 | LOC106780803 |
| 660 | MYMY4 |
| 661 | CYP46A1 |
| 662 | LRP2 |
| 663 | LRP6 |
| 664 | GCK |
| 665 | HELLS |
| 666 | IL13 |
| 667 | GC |
| 668 | PRKAA2 |
| 669 | VTN |
| 670 | ITGAL |
| 671 | MIR122 |
| 672 | FCGR2B |
| 673 | TP63 |
| 674 | GALT |
| 675 | RAD54L |
| 676 | EIF2B2 |
| 677 | UBE2L3 |
| 678 | BRDT |
| 679 | PEX6 |
| 680 | ATRIP |
| 681 | CCT7 |
| 682 | PREPL |
| 683 | STAG3 |
| 684 | TREX1 |
| 685 | PXK |
| 686 | TNIP1 |
| 687 | JAZF1 |
| 688 | BANK1 |
| 689 | MSH4 |
| 690 | IGHG1 |
| 691 | INSL6 |
| 692 | SGO2 |
| 693 | APOC4-APOC2 |
| 694 | ATRIP-TREX1 |
| 695 | SLEB3 |
| 696 | CHDS1 |
| 697 | CHDS2 |
| 698 | CHDS3 |
| 699 | CHDS4 |
| 700 | CHDS8 |
| 701 | CHDS9 |
| 702 | MCI2 |
| 703 | SLEB12 |
| 704 | SLEB13 |
| 705 | SLEB14 |
| 706 | SLEB15 |
| 707 | SLEB4 |
| 708 | SLEB5 |
| 709 | SLEB7 |
| 710 | SLEB8 |
| 711 | PRKDC |
| 712 | SPARC |
| 713 | IL32 |
| 714 | GDF2 |
| 715 | ADM |
| 716 | CAMP |
| 717 | MIR29B1 |
| 718 | S100A9 |
| 719 | CYP2E1 |
| 720 | LIPF |
| 721 | CD34 |
| 722 | PLIN1 |
| 723 | ADAMTS5 |
| 724 | MYLIP |
| 725 | H2AC18 |
| 726 | FGF2 |
| 727 | TET2 |
| 728 | G6PC2 |
| 729 | IL12B |
| 730 | ABO |
| 731 | SLC30A8 |
| 732 | CLEC1A |
| 733 | HSPA14 |
| 734 | NUDCD1 |
| 735 | PCP4 |
| 736 | PRKAA1 |
| 737 | PRKCB |
| 738 | IL37 |
| 739 | GCG |
| 740 | FASLG |
| 741 | HSPB1 |
| 742 | SAA4 |
| 743 | UCP1 |
| 744 | FTO |
| 745 | FURIN |
| 746 | MEF2C |
| 747 | PLAU |
| 748 | CSF3 |
| 749 | SERPINF1 |
| 750 | RB1 |
| 751 | MB |
| 752 | MIR590 |
| 753 | ARG2 |
| 754 | ZNF202 |
| 755 | PTPN1 |
| 756 | CH25H |
| 757 | THBS4 |
| 758 | BMP2 |
| 759 | CXCL9 |
| 760 | PINX1 |
| 761 | BMP6 |
| 762 | LEPR |
| 763 | ABCA12 |
| 764 | MYH10 |
| 765 | SPI1 |
| 766 | TNFRSF4 |
| 767 | FUT3 |
| 768 | HNF1A |
| 769 | SAMD1 |
| 770 | PALLD |
| 771 | JAG1 |
| 772 | MIR637 |
| 773 | BHMT |
| 774 | SLC9A1 |
| 775 | ADIPOR1 |
| 776 | CXCR3 |
| 777 | F9 |
| 778 | CNR1 |
| 779 | IRS1 |
| 780 | NOTCH3 |
| 781 | S100A8 |
| 782 | KCNQ1 |
| 783 | CTSB |
| 784 | PROCR |
| 785 | DGAT1 |
| 786 | NOD2 |
| 787 | IGF2 |
| 788 | GRN |
| 789 | MTNR1B |
| 790 | GAPDH |
| 791 | B2M |
| 792 | CCR7 |
| 793 | CHUK |
| 794 | CD86 |
| 795 | INSIG2 |
| 796 | NPC1L1 |
| 797 | HNF4A |
| 798 | TERF2 |
| 799 | MIR455 |
| 800 | IL6R |
| 801 | TNFSF13 |
| 802 | POSTN |
| 803 | NR4A1 |
| 804 | TNC |
| 805 | THBS2 |
| 806 | XRCC5 |
| 807 | MMP10 |
| 808 | HTRA1 |
| 809 | PDGFA |
| 810 | TAGLN |
| 811 | CCL4 |
| 812 | SCD |
| 813 | MTHFD1 |
| 814 | HBEGF |
| 815 | TEK |
| 816 | GPER1 |
| 817 | VEGFB |
| 818 | IDO1 |
| 819 | MYD88 |
| 820 | FCGR3A |
| 821 | POLR1C |
| 822 | CD44 |
| 823 | BMP4 |
| 824 | FCAMR |
| 825 | PRKAG2 |
| 826 | PROS1 |
| 827 | MIR296 |
| 828 | TSPO |
| 829 | CALR |
| 830 | PIK3C2A |
| 831 | ADH1C |
| 832 | F13B |
| 833 | CTSS |
| 834 | FDFT1 |
| 835 | SH2B3 |
| 836 | PRKAB1 |
| 837 | NECTIN2 |
| 838 | SMAD7 |
| 839 | PTGDS |
| 840 | CHST2 |
| 841 | COL8A1 |
| 842 | MIR30C1 |
| 843 | TNFSF13B |
| 844 | ATF3 |
| 845 | PIGR |
| 846 | C1QTNF9 |
| 847 | HTR2A |
| 848 | KLF5 |
| 849 | CARD8 |
| 850 | CCR1 |
| 851 | MIR204 |
| 852 | F12 |
| 853 | JAK1 |
| 854 | COL1A1 |
| 855 | IL9 |
| 856 | SLC2A4 |
| 857 | YAP1 |
| 858 | SOST |
| 859 | PLA2G2D |
| 860 | F11R |
| 861 | OSM |
| 862 | TPO |
| 863 | PLIN2 |
| 864 | ELAVL1 |
| 865 | HSPA12B |
| 866 | ITGA2B |
| 867 | SAMSN1 |
| 868 | HHIPL1 |
| 869 | AMPD1 |
| 870 | MMP7 |
| 871 | MIR222 |
| 872 | MERTK |
| 873 | MEF2A |
| 874 | MMP17 |
| 875 | ADAMTS10 |
| 876 | PPIA |
| 877 | MRTFA |
| 878 | CD274 |
| 879 | MAPK10 |
| 880 | FNDC5 |
| 881 | DMPK |
| 882 | MIR24-1 |
| 883 | FMN2 |
| 884 | WNT5A |
| 885 | CMA1 |
| 886 | ID3 |
| 887 | PPP1R3A |
| 888 | HPSE |
| 889 | PTPRC |
| 890 | LBP |
| 891 | NGF |
| 892 | SAA2 |
| 893 | C2 |
| 894 | BHMT2 |
| 895 | ATG9B |
| 896 | PDGFRB |
| 897 | CREB1 |
| 898 | SRA1 |
| 899 | GPBP1 |
| 900 | GJA1 |
| 901 | XRCC6 |
| 902 | ST3GAL4 |
| 903 | CXCL13 |
| 904 | LMNB2 |
| 905 | XK |
| 906 | TNFRSF9 |
| 907 | MCAM |
| 908 | ITGA4 |
| 909 | EPO |
| 910 | ROBO4 |
| 911 | ADAMTS3 |
| 912 | MIR424 |
| 913 | CHRNA7 |
| 914 | ACP5 |
| 915 | TF |
| 916 | TRIB3 |
| 917 | WDFY4 |
| 918 | LRRC18 |
| 919 | IL5 |
| 920 | DDAH2 |
| 921 | CCR6 |
| 922 | CACNA2D1 |
| 923 | MEFV |
| 924 | APOD |
| 925 | C1orf21 |
| 926 | GZMB |
| 927 | TBX20 |
| 928 | CCL15 |
| 929 | EP300 |
| 930 | FGF19 |
| 931 | CXCR6 |
| 932 | CAV2 |
| 933 | GSTO1 |
| 934 | ABCA7 |
| 935 | MIR185 |
| 936 | KISS1R |
| 937 | C5AR1 |
| 938 | TFAP2A |
| 939 | MIR132 |
| 940 | DNMT3A |
| 941 | CDKN3 |
| 942 | CD28 |
| 943 | MIR125B1 |
| 944 | NCF1 |
| 945 | NEU1 |
| 946 | WWTR1 |
| 947 | PVT1 |
| 948 | ADH1B |
| 949 | GRK4 |
| 950 | FOS |
| 951 | LEPQTL1 |
| 952 | CSF1R |
| 953 | EMD |
| 954 | RUNX2 |
| 955 | ABCC2 |
| 956 | M6PR |
| 957 | ITGAX |
| 958 | CAVIN1 |
| 959 | CDKN1B |
| 960 | SUCNR1 |
| 961 | CNR2 |
| 962 | MIR212 |
| 963 | PNPLA3 |
| 964 | UCP3 |
| 965 | RGS2 |
| 966 | LTB4R2 |
| 967 | MIR27A |
| 968 | F11 |
| 969 | ROCK1 |
| 970 | PTGES |
| 971 | BAX |
| 972 | MIR133A1 |
| 973 | ADAM10 |
| 974 | HMCN1 |
| 975 | ACTB |
| 976 | HS3ST1 |
| 977 | CYP2J2 |
| 978 | RECQL |
| 979 | MIR30C2 |
| 980 | AGPAT2 |
| 981 | PLCG1 |
| 982 | TNFRSF12A |
| 983 | GCH1 |
| 984 | SORCS1 |
| 985 | ANGPTL8 |
| 986 | CORIN |
| 987 | MC4R |
| 988 | HDAC1 |
| 989 | CYCS |
| 990 | TIMP4 |
| 991 | FEM1A |
| 992 | FADS1 |
| 993 | COL1A2 |
| 994 | AXL |
| 995 | NEAT1 |
| 996 | CDKN1C |
| 997 | IGFBP2 |
| 998 | AHCY |
| 999 | S1PR1 |
| 1000 | MLKL |
| 1001 | SIRT3 |
| 1002 | MIR216A |
| 1003 | BSCL2 |
| 1004 | TFPI2 |
| 1005 | UTS2R |
| 1006 | LIAS |
| 1007 | ABCA3 |
| 1008 | MIR19A |
| 1009 | HLA-DQB1 |
| 1010 | NFKBIB |
| 1011 | SMTN |
| 1012 | DKK1 |
| 1013 | GHSR |
| 1014 | IL23R |
| 1015 | ADORA2A |
| 1016 | PCSK5 |
| 1017 | PRKCE |
| 1018 | COL5A1 |
| 1019 | ABHD5 |
| 1020 | LIPK |
| 1021 | LIPM |
| 1022 | PNPLA5 |
| 1023 | LIPJ |
| 1024 | MBD2 |
| 1025 | FASN |
| 1026 | MIR141 |
| 1027 | LPXN |
| 1028 | NAT2 |
| 1029 | PDE5A |
| 1030 | GLP1R |
| 1031 | DNAH8 |
| 1032 | GPR132 |
| 1033 | HDAC4 |
| 1034 | MAOB |
| 1035 | C9orf72 |
| 1036 | MIR181B1 |
| 1037 | VCP |
| 1038 | CD80 |
| 1039 | FEN1 |
| 1040 | PIEZO1 |
| 1041 | DNMT3B |
| 1042 | CRYAA |
| 1043 | HBB |
| 1044 | CD69 |
| 1045 | RORA |
| 1046 | NTN1 |
| 1047 | ARMS2 |
| 1048 | GATA4 |
| 1049 | EZH2 |
| 1050 | FBLN1 |
| 1051 | CTSK |
| 1052 | ARG1 |
| 1053 | RPS27A |
| 1054 | BMPR2 |
| 1055 | MASP2 |
| 1056 | PRDX5 |
| 1057 | MIR663A |
| 1058 | ATP2A2 |
| 1059 | TPM2 |
| 1060 | NPM1 |
| 1061 | MIR330 |
| 1062 | BRAP |
| 1063 | PLA2G3 |
| 1064 | CTSD |
| 1065 | MIR150 |
| 1066 | LINC00113 |
| 1067 | HSPA5 |
| 1068 | ILK |
| 1069 | NFATC3 |
| 1070 | CFTR |
| 1071 | BLM |
| 1072 | LPIN1 |
| 1073 | IRF2BP2 |
| 1074 | CIDEC |
| 1075 | ABCA13 |
| 1076 | MCPH1 |
| 1077 | MT-CYB |
| 1078 | ICOS |
| 1079 | SLC2A1 |
| 1080 | LRP5 |
| 1081 | IL1R2 |
| 1082 | F2RL1 |
| 1083 | SDC4 |
| 1084 | TSLP |
| 1085 | MARS1 |
| 1086 | MTHFD1L |
| 1087 | SSTR2 |
| 1088 | CYP2C8 |
| 1089 | IGF2BP2 |
| 1090 | CDKAL1 |
| 1091 | UGT1A1 |
| 1092 | TLR1 |
| 1093 | GLA |
| 1094 | HSPB2 |
| 1095 | ADRB1 |
| 1096 | TYRO3 |
| 1097 | C1S |
| 1098 | NR4A2 |
| 1099 | IKBKG |
| 1100 | ADORA2B |
| 1101 | S1PR2 |
| 1102 | MT2A |
| 1103 | FRS2 |
| 1104 | CYP27B1 |
| 1105 | RIGI |
| 1106 | PCSK2 |
| 1107 | APOBEC1 |
| 1108 | CD79A |
| 1109 | RICTOR |
| 1110 | RHOB |
| 1111 | SLC17A4 |
| 1112 | SERPINF2 |
| 1113 | HPX |
| 1114 | TGM2 |
| 1115 | MIR30E |
| 1116 | RXRA |
| 1117 | TNFRSF10A |
| 1118 | IL19 |
| 1119 | RPA1 |
| 1120 | RECQL4 |
| 1121 | MIR423 |
| 1122 | RIPK1 |
| 1123 | FMO3 |
| 1124 | RECK |
| 1125 | CCL19 |
| 1126 | MIR200B |
| 1127 | CSN1S1 |
| 1128 | ITGAV |
| 1129 | CANT1 |
| 1130 | KCNJ2 |
| 1131 | DPH3 |
| 1132 | DDIT3 |
| 1133 | KISS1 |
| 1134 | FABP5 |
| 1135 | PGLYRP1 |
| 1136 | METTL14 |
| 1137 | PINK1 |
| 1138 | DICER1 |
| 1139 | CTNNA1 |
| 1140 | CXADR |
| 1141 | COPS5 |
| 1142 | CD55 |
| 1143 | FDX1 |
| 1144 | PTAFR |
| 1145 | LDB2 |
| 1146 | LTBP1 |
| 1147 | WNT4 |
| 1148 | TNFRSF18 |
| 1149 | IL20RA |
| 1150 | MDM2 |
| 1151 | EFEMP1 |
| 1152 | LTB |
| 1153 | PDGFC |
| 1154 | LYVE1 |
| 1155 | HBA1 |
| 1156 | MSTN |
| 1157 | FST |
| 1158 | IRF7 |
| 1159 | SERPING1 |
| 1160 | AMH |
| 1161 | CXCR5 |
| 1162 | FOXC2 |
| 1163 | THSD7A |
| 1164 | IGF2-AS |
| 1165 | MIR99A |
| 1166 | CD47 |
| 1167 | SLC11A1 |
| 1168 | CMKLR1 |
| 1169 | MIR124-1HG |
| 1170 | SPG7 |
| 1171 | CYP8B1 |
| 1172 | RAPGEF5 |
| 1173 | NEBL |
| 1174 | GLB1 |
| 1175 | MIR92A2 |
| 1176 | PCYT1A |
| 1177 | ICMT |
| 1178 | F10 |
| 1179 | GSK3B |
| 1180 | IFNGR2 |
| 1181 | KCNK1 |
| 1182 | MS4A2 |
| 1183 | TCN2 |
| 1184 | ADAM33 |
| 1185 | LGMN |
| 1186 | INSIG1 |
| 1187 | MIR19B2 |
| 1188 | CAMK2D |
| 1189 | JUP |
| 1190 | CTNNA3 |
| 1191 | AIRE |
| 1192 | lnc-KDM5D-4 |
| 1193 | G6PD |
| 1194 | SFTPD |
| 1195 | KHK |
| 1196 | HTN3 |
| 1197 | FPR2 |
| 1198 | ABCD1 |
| 1199 | IL24 |
| 1200 | CACNA1H |
| 1201 | GNAS |
| 1202 | SCNN1A |
| 1203 | SCNN1B |
| 1204 | PAX6 |
| 1205 | PRKAG1 |
| 1206 | SCNN1G |
| 1207 | PRKAB2 |
| 1208 | AMPD2 |
| 1209 | PDYN |
| 1210 | PRKAG3 |
| 1211 | RGS9 |
| 1212 | MSH3 |
| 1213 | CAPN5 |
| 1214 | MYO7A |
| 1215 | TCN1 |
| 1216 | C1QTNF1 |
| 1217 | PLEKHA1 |
| 1218 | FEM1B |
| 1219 | MIA3 |
| 1220 | NFKBIE |
| 1221 | MRPL10 |
| 1222 | OLFML2B |
| 1223 | RGS9BP |
| 1224 | JCAD |
| 1225 | TRAF1 |
| 1226 | GATA6 |
| 1227 | BCAR1 |
| 1228 | RAD51 |
| 1229 | MLH1 |
| 1230 | MRE11 |
| 1231 | POLB |
| 1232 | EXO1 |
| 1233 | ITIH4 |
| 1234 | DHX9 |
| 1235 | NHEJ1 |
| 1236 | POT1 |
| 1237 | TERF1 |
| 1238 | RAD52 |
| 1239 | RECQL5 |
| 1240 | WRNIP1 |
| 1241 | RELN |
| 1242 | SNHG16 |
| 1243 | FMOD |
| 1244 | OGN |
| 1245 | PNLIP |
| 1246 | NCOA2 |
| 1247 | VAMP3 |
| 1248 | AIF1 |
| 1249 | SELENOS |
| 1250 | KCNQ1OT1 |
| 1251 | NOTCH2 |
| 1252 | ENTPD1 |
| 1253 | CD70 |
| 1254 | MIR638 |
| 1255 | TRAF2 |
| 1256 | XIST |
| 1257 | CTF1 |
| 1258 | TAT |
| 1259 | PIWIL1 |
| 1260 | CCDC92 |
| 1261 | IL7 |
| 1262 | HDAC3 |
| 1263 | P2RX7 |
| 1264 | NFAT5 |
| 1265 | STK11 |
| 1266 | HYAL1 |
| 1267 | NUMB |
| 1268 | LCT |
| 1269 | DNAH5 |
| 1270 | ABI2 |
| 1271 | IL27 |
| 1272 | LECT2 |
| 1273 | NOX5 |
| 1274 | OR6A2 |
| 1275 | UTP20 |
| 1276 | LINC00299 |
| 1277 | MMP25 |
| 1278 | KLKB1 |
| 1279 | PDXK |
| 1280 | ACLY |
| 1281 | RXRB |
| 1282 | RXRG |
| 1283 | SLCO1B1 |
| 1284 | HCAR3 |
| 1285 | HCAR2 |
| 1286 | QPRT |
| 1287 | NNMT |
| 1288 | ADRB3 |
| 1289 | HTR1A |
| 1290 | HTR1B |
| 1291 | NR1I3 |
| 1292 | TPK1 |
| 1293 | PCYT1B |
| 1294 | ACHE |
| 1295 | PLD2 |
| 1296 | BCHE |
| 1297 | PLD1 |
| 1298 | PHOSPHO1 |
| 1299 | FNTA |
| 1300 | FNTB |
| 1301 | PLA2G4A |
| 1302 | rpoB |
| 1303 | CACNA1I |
| 1304 | CACNA1G |
| 1305 | GROUP H |
| 1306 | GHRHR |
| 1307 | DOCK4 |
| 1308 | PCNA |
| 1309 | PLCB3 |
| 1310 | CREG1 |
| 1311 | HCF2 |
| 1312 | IGHM |
| 1313 | APOBR |
| 1314 | PHLDA1 |
| 1315 | P4HA3 |
| 1316 | HSPA12A |
| 1317 | KCNN4 |
| 1318 | TTPA |
| 1319 | LINC00305 |
| 1320 | COL15A1 |
| 1321 | GPX4 |
| 1322 | IRDN |
| 1323 | MAPK9 |
| 1324 | SNN |
| 1325 | APOL3 |
| 1326 | SOHLH1 |
| 1327 | NCEH1 |
| 1328 | TTC39B |
| 1329 | ABHD2 |
| 1330 | DDHD1 |
| 1331 | BIRC3 |
| 1332 | EREG |
| 1333 | NEXNAS1 |
| 1334 | ADRA1B |
| 1335 | CYP24A1 |
| 1336 | ITGB7 |
| 1337 | IRF1 |
| 1338 | PLXND1 |
| 1339 | MAB21L2 |
| 1340 | OSCAR |
| 1341 | KALRN |
| 1342 | LECT1 |
| 1343 | PRKCH |
| 1344 | LPIN2 |
| 1345 | LPIN3 |
| 1346 | ADAM15 |
| 1347 | LYPLA1 |
| 1348 | KIF20A |
| 1349 | NUDT6 |
| 1350 | HAL |
| 1351 | MIR125B2 |
| 1352 | SCUBE1 |
| 1353 | TNFAIP8 |
| 1354 | CPS1 |
| 1355 | MIR217 |
| 1356 | APOPT1 |
| 1357 | TXNDC5 |
| 1358 | PDE1A |
| 1359 | MTRNR2 |
| 1360 | TMBIM1 |
| 1361 | YJEFN3 |
| 1362 | HOXAAS3 |
| 1363 | INO80D |
| 1364 | BANCR |
